# Magnitude of Anxiety and Depression and Associated Factors among Palliative Care Patients with Cancer at Tikur Anbessa Specialized Hospital, Ethiopia

**DOI:** 10.4314/ejhs.v32i2.14

**Published:** 2022-03

**Authors:** Bantalem Tilaye Atinafu, Tefera Mulugeta Demlew, Fetene Nigussie Tarekegn

**Affiliations:** 1 Department of Nursing, College of Health Science, Debre Berhan University, Debre Berhan, Ethiopia; 2 School of Nursing and Midwifery, College of Health Sciences, Addis Ababa University, Addis Ababa, Ethiopia

**Keywords:** Anxiety, depression, palliative, cancer, magnitude, Ethiopia

## Abstract

**Background:**

Little is known regarding the severity of anxiety and depression among palliative care patients with cancer. As a result, this study aimed to assess the magnitude of anxiety and depression and its associated factors among palliative care patients with cancer.

**Methods:**

A cross-sectional study was conducted at the Black Lion Specialized Hospital oncology center on palliative care patients with cancer who had follow up. Interviews and chart reviews were used. Descriptive statistics and Chi-square were done. Multivariate analysis was done.

**Result:**

A total of 171 palliative care patients with cancer were involved in the study. The magnitude of anxiety and depression was found at 64.9% and 47.4%, respectively. Those palliative care patients with cancer whose age >64 years (AOR: 7.1; CI: 1.59–68.0; P =0.029), unable to write and read (AOR: 0.2; CI: 0.03–0.73; P = 0.017), secondary school (AOR: 0.3; CI: 0.11–0.83; P = 0.022) were significant factors for anxiety. Breast cancer (AOR: 0.1; CI: 0.01–0.85; P = 0.021), surgery plus radiation (AOR: 0.2; CI: 0.02–0.91: P=0.024) others (radiation and surgery (AOR: 0.1; CI: 0.02–0.8: P = 0.036) were found to be significant factors for depression.

**Conclusion:**

The magnitude of anxiety and depression was 64.9% and 47.4%, respectively. Greater than 64-year-old age, unable to write and read secondary school were significant factors for anxiety. Breast cancer, surgery plus radiation, others (radiation and surgery) were found to be significant factors for depression.

## Introduction

Cancer is the second leading cause of disease globally which accounts for 9.6 million deaths (1). WHO estimated that cancer cases increase at an alarming rate. Of which, about 81% of new cancer cases occur in low- and middle-income countries (2). Consequently, cancer imposes a substantial problem on physical, emotional, psychological, and financial dimensions (3).

Depression and anxiety are the most common problems encountered among patients with cancer (4). About 20% and 10% of patients with cancer are affected by depression and anxiety, which is higher than the general population, accounting for 5% and 7% respectively (5). The magnitude of depression among patients with cancer ranges from 5% to 60%. Whereas, 37% and 55.7% of terminally ill patients with cancer had anxiety and depression respectively (6). In addition, the burden of depressive and anxiety disorders among palliative care patients with cancer was shown as 20.7% and 13.9% respectively (7). Evidence from Australia showed that about 45.8% of palliative cancer patients had depression whereas 36.9% had anxiety (8). Hence, anxiety and depression were common problems among palliative care patients with cancer (9).

The spectrums of anxiety and depression disorders are complicated by symptoms burden that makes diagnosis and treatment more difficult (10). Patients at an advanced stage of cancer develop depression, which causes a decrease in motivation towards self-care, poor adherence to treatment interventions, the difficulty of cooperating with a plan of care. Anxiety and depression among palliative care patients with cancer had a negative significant effect on survival outcomes, quality of treatment, and quality of life (11, 12).

Experience shows that sex, educational level, residence, occupation social support and monthly income, family history, type of cancer were found to be associated factors for anxiety and depression in palliative care patients with cancer (13–16).

In Ethiopia, cancer accounts for about 5.8% of mortality, and the incidence was over 60,960 cases annually. Of those diagnosed patients with cancer, about 80% of patients with cancer were diagnosed at an advanced stage. However, the national cancer program had a plan to improve palliative care services; it is inclined to strategically link palliative services to early detection, prevention, and treatment services. Assessment of the prevalence of the psychiatric problem is vital for the improvement of the quality of life of palliative care patients with cancer (17). However, the emphasis is not given to frequently occurring anxiety and depressions in palliative care patients. Thus, little is known about the magnitude of anxiety and depression among palliative care patients with cancer. Thus, this study aimed to assess the magnitude of anxiety and depression and associated factors among palliative care patients with cancer.

## Methods and Materials

**Study design, period, and setting**: An institution-based cross-sectional study was conducted in the oncology center of Black Lion Specialized Hospital (BLSH), Addis Ababa, Ethiopia, from March 9 to April 09/ 2020. The oncology center is housed in a separate building on the hospital grounds, and a newer building has been built in Lideta Kifle Ketema. The center gives service to more than 60,000 palliative care patients with cancer settings annually and there were 5 clinical oncologists, 7 radiotherapists, 9 oncology nurses, and 18 nurses working at the oncology center. The oncology center consists of three floors: the clinic with three examination rooms, physicians' offices, waiting rooms, a pharmacy, and radiation vaults; a floor for the inpatient ward; and a floor for meeting rooms (18).

**Population**: The source population was all patients with cancer who had a follow-up in the palliative care setting of the Black Lion's specialized hospital oncology center. The study population was patients with cancer who had a follow-up in a palliative care setting within the study period at the oncology center of BLSH. Patients under the age of 18 and those with hearing and speaking difficulties were excluded.

**Sample size determination**: First, the number of patients with cancer who had a follow-up in a palliative care setting was determined. There were approximately 54 patients with cancer who had follow-ups every week. Then, the study took all those patients with cancer who had a follow-up within a month. About 216 patients with cancer who had a follow-up in the palliative cancer setting were selected. Among 216 patients with cancer, 35 patients were less than 18 years old, and 10 patients with cancer had the hearing and speaking difficulties. Finally, 171 study subjects were involved in the study.

**Data collection methods and procedures**: First, supervisors and data collectors were selected. The coordinator nurse in the oncology center carried out the supervisory activity. About 3 BSc oncology nurses, 1 MSc student, and 1 MSc oncology nurse were involved in the data collection. Before data collection, training was given to the data collectors. The data were collected using a structured interview questionnaire that consisted of sociodemographic factors, clinicopathological factors, and hospital anxiety and depression scale. A chart review was done for palliative care patients with cancer because of symptom burden and unable to clearly state the type of cancer, treatment received, length of diagnosis, and comorbid conditions. The hospital anxiety and depression scale (HADS) found a reliable instrument for detecting the state of depression and anxiety in the medical hospital setting (19). HADS had anxiety and depression sub-scales to screen the frequency of depression and anxiety symptoms. It is a 4-point scale that ranges from 0 to 3. The total score takes values from 0 to 21. The higher score refers to depression and anxiety (20). The cutoff point for each sub-scale for depression and anxiety was rated as scores 0 to 8 and 8 to 21 considered as presence and absence of symptoms respectively (21). The HADS has been widely used as a screening tool for psychiatric morbidity in general hospital patients; also its validity to screen medical patients has been confirmed in Ethiopia. The internal consistency for anxiety, depression, and full scale (HADS) was 0.78, 0.76, and 0.87, respectively (22).

**Data processing and analysis**: The collected data were cleaned, checked, edited, coded, and then entered into Epi-data version 4.2, and exported into SPSS Version 25 for analysis. Descriptive statistics and chi-square tests were done. Independent variables were linearly related to the log of odds. The observations were assumed to be independent of each other, the dependent variables were assumed to be binary and the sample size was appropriate based on the number of patients who had to follow up in a palliative cancer setting.

In Bivariate logistic regression, those independent variables which were fitted to the bivariate logistic regression p-value of less than or equal to 0.25 were entered into the multivariate analysis. Multivariate logistic regression was done at a 0.05 level of significance. The p-value of less than 0.05 in the multivariate analysis was considered to be statistically significant. The results of the study were expressed through texts, tables, and figures.

**Ethical consent**: Ethical clearance was obtained from the ethical research committee department of nursing, School of Nursing and Midwifery, Addis Ababa University. Approval was obtained for data collection from the medical director and cancer treatment center focal person of Black Lion Specialized Hospital. Written informed consent was obtained from the study participants. Confidentiality of the information was kept throughout the study by excluding patient names as identification from the data collection form. To keep confidentiality all collected data were coded and locked in a separate room before being entered into the computer. After entering the computer, the data were locked by password, and the data haven't been disclosed to any person other than the investigators. Patients with anxiety and depression were recommended to be linked to the psychiatry clinic.

## Results

**Socio-demographic characteristics of the study participants**: A total of 171 study participants have participated in the study. Regarding the age of study participants, 78 (45.6%) of the participants were aged between 44–64 years, and the mean age was 47.03±13.6SD years old. About 37(21.6%) of study participants had a monthly income of less than or equal to 500 birrs and the average income was 2915.6 Ethiopian birr (Table 1).

**Table 1:** Sociodemographic characteristics of study participants among palliative care patients with cancer at Black Lion Specialized Hospital Oncology Center, Addis Ababa, 2020(n=171)

Variable	Category	Frequency (n)	Percent (%)
**Gender**	Male	54	31.6
	Female	117	68.4
**Age**	< 44	75	43.9
	44–64	78	45.6
	>64	18	10.5
**Marital status**	Single	29	17.0
	Married	117	68.4
	Others^1^	25	14.6
**Residence**	Urban	126	73.7
	Rural	45	26.3
**Monthly income**	<=500	37	21.6
	501–1000	29	17.0
	>=1001	105	61.4
**Educational status**	Can't read and write	22	12.9
	Primary	62	36.3
	Secondary	41	24.0
	College and university	46	26.9
**Occupational status**	Unemployed	4	2.3
	Employed	63	36.8
	Housewife	46	26.9
	Farmer	25	14.6
	Daily labourer	17	9.9
	Others^2^	16	9.4

N.B: **others^1^**:- Divorced, widowed; **others^2^**:- merchant, pension, student

**Clinico-pathological characteristics of the study participants**: Among the study participants, about 62 (36.3%) were breast cancer patients. The mean length of diagnosis was 21.8 ± 20.98 SD months.

About 56 (32.7%) of the study participants had comorbid conditions. Regarding the treatment received, 80 (46.8%) of study participants received chemotherapy (Table 2).

**Table 2:** Clinico-pathological characteristics of study participants among palliative care patients with cancer at Black Lion Specialized Hospital Oncology Center, Addis Ababa, 2020(n=171)

Variable	Category	Frequency(n)	Percent (%)
**Tumour type**	Lung cancer	14	8.2
	Breast cancer	62	36.3
	Colorectal cancer	38	22.2
	Cervical cancer	13	7.6
	Sarcoma	11	6.4
	Others*	33	19.3
**Duration of**	<12 months	64	37.4
**diagnosis**	>=12 months	107	62.6
**Comorbidity**	Yes	56	32.7
	No	115	67.3
**Intervention**	Chemotherapy	80	46.8
	Surgery plus chemo	44	25.7
	Radiation +surgery+ chemo	22	12.9
	Surgery plus radiation	15	8.8
	Others**	10	5.8

N.B: Others*-lymphoma, prostate Ca, Testicular Ca, pancreatic Ca, leukemia Ca, endometrial Ca, vulvar Ca, the skin ca, laryngeal Ca, nasopharyngeal Ca, orbital Ca, oral Ca; others**: radiation, surgery

**Magnitude of anxiety and depression among palliative care patients with cancer**: The magnitude of anxiety among palliative care patients with cancer was found to be 111(64.9%) (95% CI: 56.7–72.5%). Whereas the magnitude of depression was found to be 81(47.4%) (95% CI: 40.0–54.4%).

The prevalence of anxiety among study participants aged from 44 to 64 was 48 (43.2%). The magnitude of anxiety was statically different among age groups with a p-value of 0.021. Regarding the type of cancer, about 36 (32.4%) of anxiety was reported from patients with breast cancer. The prevalence of anxiety among patients who had received chemotherapy was 56 (50.5%).

Among study participants, 52 (64.2%) of depression was found among female patients with cancer. The magnitude of depression among employed study participants was 25 (30.9%). There was a statistical difference in the prevalence of depression among occupations with a p-value of 0.015. About 44 (54.3%) of depression was found among palliative care patients with cancer whose length of diagnosis was greater than or equal to 12 months (Table 3).

**Table 3:** Association of independent variables with anxiety and depression among palliative care patients with cancer at Black Lion Specialized Hospital Oncology Center, Addis Ababa, 2020(n=171)

Variable	Category	Anxiety			Depression		
		Yes n (%)	No n (%)	P-value	Yes n(%)	No n(%)	p-value
**Gender**	Male	38(34.2)	16(26.7)	0.31	29(35.8)	25(27.8)	0.260
	Female	73(65.8)	44(73.3)		52(64.2)	65(72.2)	
**Age**	<44	46(41.5)	29(48.3)	0.021	33(40.7)	42(46.7)	0.082
	44–64	48(43.2)	30(50.0)		35(43.2)	43(47.8)	
	>64	17(15.3)	1(1.7)		13(16.1)	5(5.5)	
**Marital status**	Single	20(18.0)	9(15.0)	0.342	15(18.5)	14(15.6)	0.863
	Married	72(64.9)	45(75.0)		54(66.7)	63(70.0)	
	Others^1^	19(17.1)	6(10.0)		12(14.8)	13(14.4)	
**Residence**	Urban	79(71.2)	47(78.3)	0.310	62(76.5)	64(71.1)	0.421
	Rural	32(28.8)	13(21.7)		19(23.5)	26(28.9)	
**Monthly**	<=500	28(25.2)	9(15.0)	0.202	16(19.8)	21(23.3)	0.851
**income**	501–1000	20(18.0)	9(15.0)		14(17.3)	15(16.7)	
	>=1001	63(56.8)	42(70.0)		51(62.9)	54(60.0)	
**Educational**	Can't read and write	17(15.3)	5(8.3)	0.015	11(13.6)	11(12.2)	0.706
**status**	Primary	41(37.0)	21(35.0)		29(35.8)	33(36.7)	
	Secondary	30(50.0)	11(18.3)		22(27.2)	19(21.1)	
	College and university	23(20.7)	23(38.4)		19(23.4)	27(30.0)	
**Occupational**	Unemployed	3	1	0.241	3(3.7)	1(1.1)	0.015
**status**	Employed	35(31.5)	28(46.6)		25(30.9)	38(42.2)	
	House wife	30(27.1)	16(26.6)		21(25.9)	25(27.8)	
	Farmer	18(16.2)	7(11.6)		12(14.8)	13(14.5)	
	Daily labourer	11(9.9)	6(10.0)		6(7.4)	11(12.2)	
	Other^2^	14(23.3)	2(3.2)		14(17.3)	2(2.2)	
**Tumour type**	Lung cancer	11(9.9)	3(5.0)	0.079	10(12.3)	4(4.4)	0.039
	Breast cancer	36(32.4)	26(43.0)		21(25.9)	41(45.6)	
	Colorectal cancer	30(27.1)	8(13.0)		20(24.7)	18(20.0)	
	Cervical cancer	6(5.4)	7(11.7)		5(6.2)	8(8.9)	
	Sarcoma	9(8.1)	2(3.3)		8(9.9)	3(3.3)	
	Others*	19(17.1)	14(74.0)		17(21.0)	16(17.8)	
**Duration of**	<12 months	45(40.5)	19(31.7)	0.252	37(45.7)	27(30.0)	0.340
**diagnosis**	>=12 months	66(59.5)	41(68.3)		44(54.3)	63(70.0)	
**Comorbidity**	Yes	38(34.2)	18(30.0)	0.573	26(32.1)	30(33.3)	0.864
	No	73(65.8)	42(70.0)		55(67.9)	60(66.7)	
**Intervention**	Chemotherapy	56(50.5)	24(40.0)	0.047	45(55.6)	35(38.9)	0.035
	Surgery plus chemo	30(27.0)	14(23.3)		21(25.9)	23(25.6)	
	Radiation + surgery + chemo	12(10.8)	10(16.7)		9(11.1)	13(14.4)	
	Surgery plus radiation	7	8		4(4.9)	11(12.2)	
	Others**	6	4		2	8	

N.B: **others^1^**:- Divorced, widowed; **others^2^others^2^**:- merchant, pension, student; Others*:-lymphoma, prostate Ca, Testicular Ca, pancreatic Ca, leukemia Ca, endometrial Ca, vulvar Ca, the skin ca, laryngeal Ca, nasopharyngeal Ca, orbital Ca, oral Ca; others**:- radiation, surgery

**Associated factors of anxiety among palliative care patients with cancer**: In the bivariate analysis, age, monthly income, educational status, type of tumor, and intervention (radiation plus surgery, and surgery and radiation) were fitted for anxiety at a p-value of less than or equal to 0.25. In multivariate analysis, age greater than 64 years old had a positive significant effect on anxiety; being unable to write and read, and secondary school had negative significant factors for anxiety.

Those palliative care patients with cancer whose age was greater than 64 years old were 7.1 times more likely to develop anxiety than patients aged less than 44 years old (AOR: 7.1; CI: 1.59–68.0; P = 0.029). Those patients who had completed secondary school were 70.0% less likely to develop anxiety than patients who had finished college and university (AOR: 0.3; CI: 0.11–0.83; P= 0.022) (Table 4).

**Table 4:** Bivariate and multivariate analysis of anxiety and depression among palliative care patients with cancer in Tikur Anbessa Specialized Hospital Oncology center, Addis Ababa Ethiopia (n=171)

Variable	Category	Anxiety			Depression		
		Bivariate	Multivariate	P value	Bivariate	Multivariate	P value
**Gender**	Male	1.4(0.71–2.86)	-		1.4(0.76–2.78)	-	
	Female	1					
**Age**	<44	1	1		1	1	
	44–64	1.0(0.51–1.90)	1.2(0.56–2.65)	0.619	1.0(0.51–1.82)	0.9(0.94–2.01)	0.993
	>64	10.7(2.1–72.0)	7.1(1.59–68.0)	0.029	3.3(0.09–9.03)	4.0(0.09–8.4)	0.300
**Marital status**	Single	1	-		1	-	
	Married	0.7(0.21–2.35)			0.8(0.55–2.82)		
	Others	1.4(0.58–3.32)			0.9(0.39–3.39)		
**Residence**	Urban	0.68(0.32–1.43)	-		1.3(0.67–2.63)	-	
	Rural	1					
**Monthly**	<=500	2.1(0.20–5.12)	0.6(0.21–1.97)	0.372	0.8(0.58–2.64)	-	
**income**	501–1000	1.5(0.49–4.93)	1.2(0.28–3.62)	0.473	1.0(0.44–2.30)		
	>=1001	1	1		1		
**Educational**	Can't read and write	0.3(0.07–0.89)	0.2(0.03–0.73)	0.017	0.7(0.25–1.95)	-	
**status**	Primary						
	Secondary	0.5(0.06–5.37)	0.35(0.12–1.03)	0.061	0.8(0.37–1.73)		
	College and	0.4(0.03–0.9)	0.3(0.11–0.83)	0.022	0.6(0.26–1.42)		
	university	1	1		1		
**Occupational**	Unemployed	1				1	
**status**	Employed	0.4(0.24–24.3)	-		0.2(0.16–34.9)	0.4(0.76–17.2)	0.079
	House wife	0.6(0.15–16.6)			0.3(0.02–50.9)	0.9(0.45–16.8)	0.155
	Farmer	0.8(0.10–13.2)			0.3(0.09–40.9)	0.6(0.47–22.8)	0.169
	Daily labourer	0.6(0.14–19.3)			0.2(0.02–40.5)	0.8(0.5–32.8)	0.062
	Other	2.3(0.03–6.40)			2.3(2.1–76.4)	1.4(0.06–31.4)	0.935
**Tumour type**	Lung cancer	1	1		1	1	
	Breast cancer	0.4(0.07–10.4)	0.6(0.50–11.7)	0.215	0.2 (0.04–0.78)	0.1(0.01–0.85)	0.021
	Colorectal cancer	1.0(0.22–4.36)	1.9(0.12–3.28)	0.566	0.4(0.09–8.45)	0.6(0.39–6.69)	0.544
	Cervical cancer	0.2(0.09–22.9)-	0.1(0.08–28.9)	0.127	0.09(0.07–20.0)	0.8(0.29–11.5)	0.410
	Sarcoma	0.81(0.11–5.98)	0.6(0.06–5.94)	0.751	1.1(0.16–5.46)	4.0(0.04–6.5)	0.616
	Others^3^	0.4(0.03–11.5)	0.1(0.50–12.6)	0.315	0.4(0.61–9.04)	0.9(0.46–8.37)	0.433
**Duration of**	<12 months	1.5(0.76–2.85)	-		1.9(1.0–3.77)	-	
**diagnosis**	>=12 months	1			1		
**Comorbidity**	Yes	1.2(0.61–2.39)	-		0.9(0.49–1.79)	-	
	No				1		
**Intervention**	Chemotherapy	1	1		1	1	
	Surgery plus chemo	0.9(0.49–2.41)	0.4(0.05–3.74)	0.167	0.7(0.67–2.94)	0.3(0.06–3.27)	0.604
	Radiation+surgery + chemo	0.5(0.04–5.10)	0.06(0.03–6.05)	0.493	0.5(0.1–4.84)	0.9(0.44–3.86)	0.738
	Surgery plus radiation	0.4(0.08–8.18)-	0.8(0.63–9.63)	0.260	0.3(0.03– 0.89)	0.2(0.02–0.91)	0.024
	Others^4^	0.6(0.40–6.01)	0.7(0.57–12.8)	0.218	0.2(0.02–0.79)	0.1(0.02–0.87)	0.036

**N.B: not included because those variables were not fitted at p≤0.25; others^1^**; Divorced, widowed; **others^2^**; merchant, pension, student; **Others^3^**; lymphoma, prostate Ca, Testicular Ca, pancreatic Ca, leukemia Ca, endometrial Ca, vulvar Ca, the skin ca, laryngeal Ca, nasopharyngeal Ca, orbital Ca, oral Ca; **others^4^**; radiation, surgery

**Associated factors of depression among palliative care patients with cancer**: In bivariate analysis, age, occupational status, tumor type, and intervention (radiation plus surgery, surgery, and radiation) were fitted for depression outcome variable at a p-value less than or equal to 0.25. Breast cancer, radiation plus surgery as well as others (radiation and surgery) receiving patients were found to be negative significant factors for depression in multivariate analyses.

Those palliative care patients with breast cancer were 90% less likely to develop depression than patients with lung cancer (AOR: 0.1; CI: 0.01–0.85; P = 0.021). Those Palliative care patients with cancer who received radiation plus surgery were 80.0% less likely to develop depression than those who received chemotherapy (AOR: 0.2; CI: 0.02–0.91; P=0.024) (Table 4).

## Discussion

In this study, the magnitude of anxiety among palliative care patients with cancer was 64.9 % (95% CI: 56.7–72.5%). This is also higher than study conducted in New York [7.6%] (23), in China among patients with breast cancer [31.65%] (24),[7.1%] (25), In Malaysia [31.7%] (26), Singapore [9.5%] (27), Korea [30.0%] (28) and Portuguese [23.7%] (29). Whereas, the magnitude of depression among palliative care patients with cancer was 47.4% (95% CI: 40.0–54.4%). This finding was higher than the study done in Michigan [32.4%] (30), Athens among patients with breast cancer [38.2] (31), Malaysia [22.0%] (26), Singapore [16.8%] (27), and Portuguese [24.4%] (29). This discrepancy could be attributable to a difference in psychological intervention or consultation and study populations, the tool used for screening, way of analysis, and socio-demographic variations. Evidence from the Portuguese revealed that there was a strong family bonding culture. These families bonding enable the clients to express their feelings and thought, and discuss the positive aspect of life. Therefore, it could reduce depression and anxiety. Also, the previous studies have investigated its burden among all cancer and specific cancer types. But, the current study focused on palliative care patients. Because being diagnosed with advanced cancer is often associated with an increased level of pain and a higher level of existential concerns related to fear of death or fear of being uncontrolled when the financial and psychological cost of care becomes intolerable to friends and relatives.

Contrariwise, the finding of depression in this current study was lower than studies conducted in Pakistan [62.7%] (32), and [87.4%] (33). Besides, the current magnitude of anxiety was lower than the study done in Pakistan [88.5%] (33). This could be due to biological factors and the physiological response of the body towards cancer and its treatment. However, the finding of depression was in line with a study done in Nepal [49.2]. This was perhaps due to a study in Nepal being done among patients with cancer who were on treatment. Thus, treatment-related side effects patients were imposed to develop depression.

Besides, the magnitude of anxiety in the present study was higher than studies conducted in Australia and wales, [20.0%] (34), Canada [13.9%] (35). The current finding of depression was also higher than the study conducted in Canada among palliative care patients with cancer [20.7%] (35). This difference was due to better health service interventions and psychological counseling delivered to palliative care patients with cancer in high-income countries.

This finding was higher than the study conducted in African countries such as Nigeria with a prevalence of anxiety and depression 36.9% and 31.6% respectively (36). However, this finding was in line with the studies conducted in Rwanda [67.7%] the prevalence of anxiety (37) and Kenya with a 42.0% prevalence of depression (38). This was perhaps due to differences in study participants' characteristics. Most of the study participants were stage III and stage IV cancer cases; this made the finding in line with the current finding. Whereas, other studies were done among the general population living with cancer. The current finding of depression was lower than the study conducted in Gondar and Felege Hiwot referral hospitals [70.89%] (39).

The discrepancy could be attributable to the difference in the study populations in terms of types of cancer, the tool used for screening, or other socio-demographic variations and severity of depression. In the previous studies, Beck's depression inventory (BDI-II) and the fear of progression (FOP 12) (40). There was a psychometric agreement comparing HADS-D ≥8 with PHQ-9 ≥12 for providing equalized anxiety and depression-related information (41). However, the study in Gondar and Felege Hiwot referral hospital has used PHQ-9 cut of a score greater and equal to 10 scores. This could estimate the higher prevalence of the HADS score greater than or equal to 8 scores.

Moreover, this inconsistency was perhaps due to the difference in assessment tools across studies. Some studies use hospital anxiety and depression scale, DSM-IV, DSM-V, physical health questionnaire. Moreover, a different approach in the case of cut-off point used the treatment setting, availability of treatment facilities among developed countries regarding psychological dimensions. From the previous studies, the current finding is higher from the overall cancer case and specific to palliative care patients with cancer. In the current study, the prevalence of anxiety and depression among patients with colorectal cancer was 78.9% and 52.6% respectively. This finding was lower than the study conducted among gastric cancer patients due to better treatment intervention offered and accessibility of comprehensive therapy in China (42, 43). This could be because less attention was given to anxiety and depression among patients with advanced cancer. Besides, the current pandemic novel Corona Virus-19 precipitates psychological problems among comorbid conditions. The recovery rate of comorbid patients who had COVID-19 was lower than the noncomorbid condition. Therefore, those patients with cancer who need intensive follow-up and care lose hope and were uncooperative with the given pharmacologic and non-pharmacologic interventions (44).

The current study revealed that age was found to be a significant factor for anxiety and depression among palliative care patients with cancer. This is similar to a study conducted in Iran (45). This is because old age increases the duration of disease, the high probability of cancer metastasis, and more disability, and these conditions increase anxiety and depression in older patients. In normal instances, elders experience life-stressing events such as a drop in socioeconomic status with retirement and physiological deterioration. The presence of medical conditions made them more stressed (46, 47).

Those palliative care patients with cancer who can't read and write and secondary school are found as a significant factor for anxiety. This finding was similar to previous studies conducted in Nigeria (16), Rwanda (37). However, another study showed that lower education is associated with a positive association with anxiety and depression (48). This is perhaps due to being informed about the nature of the disease, the type of treatment, and its side effects which alter the level of anxiety and anxiety, and depression across the different sections of the population. The other possible explanation could be the presence of a greater number of higher-educated palliative care patients. Despite appropriate medical evaluation and reassurance, preoccupation with fears of having a serious disease-based misinterpretation, informed with the complication outcomes of the disease precipitates anxiety and depression (49).

In this study, those Palliative care patients with cancer who received radiation plus surgery were 80.0% less likely to develop depression than those who received chemotherapy. This is perhaps due to the familiarity of patients with radiation plus surgery treatment. This could lower the anxious situation. However, chemotherapy treatment-related side effects may present even if the client understands the nature of the disease and pharmacologic mechanisms of the treatment. As evidence revealed that corticosteroids, interferonalpha, interleukin-2, gonadotropin-releasing hormone agonists, mefloquine, progestin-releasing implanted contraceptives, and propranolol therapies induce psychiatric problems (50). Evidence showed that the burden of care given and health care cost among cancer patients changes over time, and had a substantial effect on financial loss and loss of opportunities (51).

The limitation study of the study includes; the study was cross-sectional, which cannot determine the causation among palliative care patients with cancer; the study was quantitative (it didn't incorporate the parameters addressed qualitatively), and anxiety and depression were classified as presence and absence, these could overestimate the prevalence of anxiety and depression.

In conclusion, the magnitude of anxiety and depression among palliative care patients with cancer was 64.9% and 47.4%, respectively. Age greater than 64 years old had a positive significant effect on anxiety; unable to write and read, and secondary school had negative significant factors for anxiety. Breast cancer, radiation plus surgery, and others (radiation and surgery) receiving patients were found to be negative significant factors for depression. Emphasis should be given to elderly patients and those who receive chemotherapy. The hospital should also perform a regular assessment. The Black Lion Specialized Hospital, Saint Amanuel Mental Specialized Hospital, and School of Psychology Addis Ababa University should work jointly on psycho-oncology issues.
